# Crystal structure of (*E*)-1-(3-benzyl-5-phenyl-1,3-thia­zol-2-yl­idene)-2-[(*E*)-1,2,3,4-tetra­hydro­naphthalen-1-yl­idene]hydrazin-1-ium bromide

**DOI:** 10.1107/S2056989021002863

**Published:** 2021-03-23

**Authors:** Shaaban K. Mohamed, Sahar M. I. Elgarhy, Alaa A. Hassan, Güneş Demirtaş, Joel. T. Mague, Youssef Ramli

**Affiliations:** a Chemistry and Environmental Division, Manchester Metropolitan University, Manchester M1 5GD, England; b Chemistry Department, Faculty of Science, Minia University, 61519 El-Minia, Egypt; c Chemistry Department, Faculty of Science, Assuit University, Egypt; d Ondokuz Mayis University, Faculty of Arts and Sciences, Department of Physics, 55139, Samsun, Turkey; e Department of Chemistry, Tulane University, New Orleans, LA 70118, USA; f Laboratory of Medicinal Chemistry, Faculty of Medicine and Pharmacy, Drug, Sciences Research Center, Mohammed V University in Rabat, Morocco

**Keywords:** crystal structure, di­hydro­naphthalene, thia­zole, hydrazinium salt, hydrogen bond

## Abstract

In the title mol­ecular salt, C_26_H_24_N_3_S^+^·Br^−^, the dihedral angles between the thia­zole ring and its attached phenyl and benzoyl rings are 54.81 (7) and 85.51 (7)°, respectively. In the crystal, ion pairs are linked by C—H⋯Br and N—H⋯Br hydrogen bonds and are connected into helical chains extending along the *c*-axis direction by weak, electrostatic S⋯Br^−^ inter­actions. A Hirshfeld surface analysis was performed, which showed the dominant role of H⋯H contacts (51.3%).

## Abstract 2

In the crystal of the title mol­ecular salt, ion pairs are linked by C—H⋯Br and N—H⋯Br hydrogen bonds, which are connected into helical chains extending along the *c*-axis direction by weak, electrostatic S⋯Br^−^ inter­actions.

## Chemical context   

**Figure 1:**
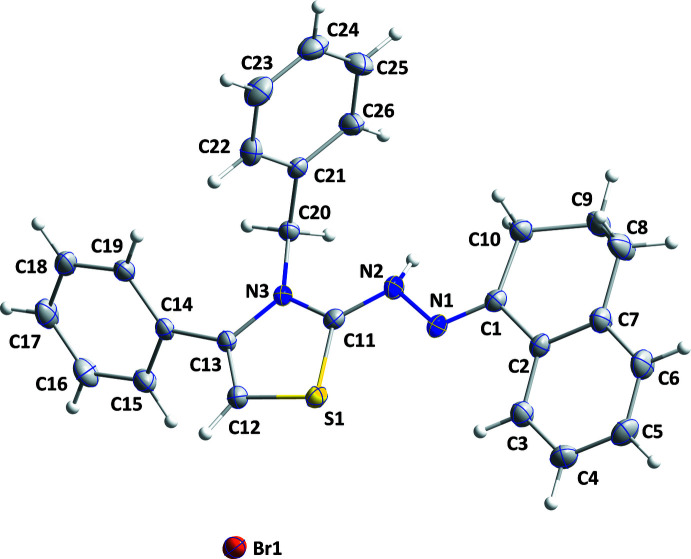
The title mol­ecule showing 50% probability ellipsoids.

Thia­zoles are a class of heterocyclic compounds found in many biologically active drugs such as sulfa­thia­zol (anti­microbial drug), ritonavir (anti­retroviral drug), abafungin (anti­fungal drug) and tiazofurin (anti­neoplastic drug) (Siddiqui *et al.*, 2009 ▸). Other compounds containing the thia­zole or thia­zolyl moiety show numerous biological activities such as anti­microbial and anti­fungal (Vasu *et al.*, 2013 ▸), anti-inflammatory (Singh *et al.*, 2008 ▸), anti­cancer (Luzina *et al.*, 2009 ▸), anti­hypertensive (Turan-Zitouni *et al.*, 2000 ▸), anti-HIV (Rawal *et al.*, 2008 ▸), anti­convulsant (Satoh *et al.*, 2009 ▸) and anti­diabetic properties (Iino *et al.*, 2009 ▸). As with many biologically active mol­ecules, the mol­ecular conformation adopted may have a significant effect on the activity which prompted an examination of the crystal structure of the title salt, C_26_H_24_N_3_S·Br, **I** (Fig. 1 ▸).
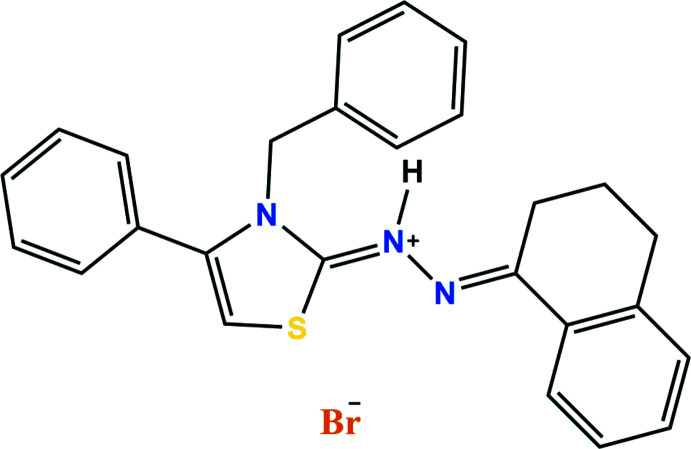


## Structural commentary   

As expected, the C11/C12/C13/N3/S1 thia­zole ring in **I** is almost planar (r.m.s. deviation = 0.0056 Å) and the mean planes of the C14–C19 and C21–C26 benzene rings are inclined to this plane by 54.81 (7) and 85.51 (7)°, respectively. The dihedral angle between the mean planes of the thia­zole and C2–C7 rings is 13.1 (1)°. A puckering analysis of the C1/C2/C7–C10 ring yielded the parameters *Q* = 0.499 (3) Å, θ = 58.6 (3)° and φ = 225.6 (3)°, indicating a half-chair conformation.

## Supra­molecular features   

**Figure 2:**
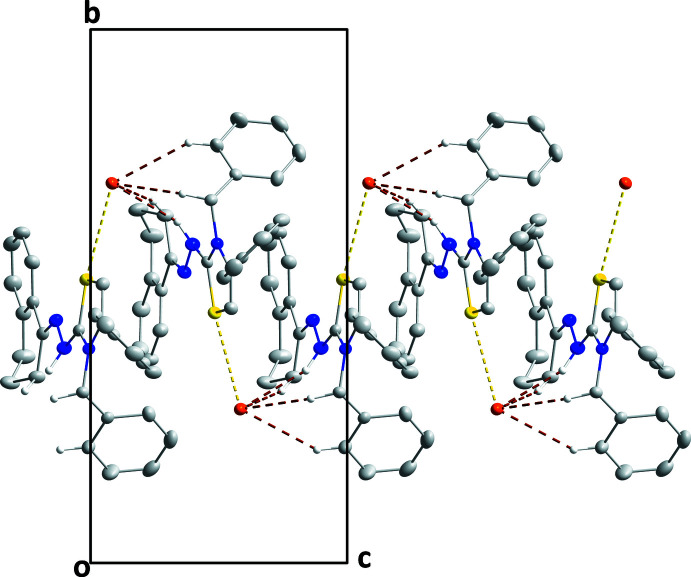
Detail of a supra­molecular chain viewed along the *a*-axis direction with C—H⋯Br and N—H⋯Br hydrogen bonds depicted by brown dashed lines. The short Br⋯S contact is depicted by a yellow dashed line.

**Figure 3:**
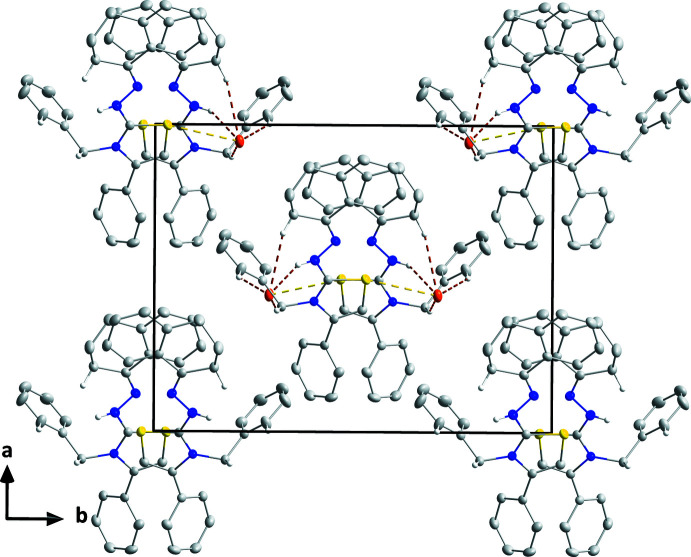
Packing seen along the *c*-axis direction giving an end view of the chains. Inter­molecular inter­actions are depicted as in Fig. 2 ▸.

In the crystal, the S1⋯Br1 distance of 3.5017 (7) Å is some 0.15 Å less than the sum of the van der Waals radii and likely represents an electrostatic inter­action between the two atoms since S1 is near to the cationic charge. Over 200 structures having S⋯Br^−^ contacts of this length or shorter are present in the Cambridge Structural Database, two examples being reported by Auffinger *et al.* (2004 ▸) and Thompson & Richardson (1977 ▸). This inter­action, together with the N2—H2⋯Br1, C10—H10*B*⋯Br1, C20—H20*B*⋯Br1 and C26—H26⋯Br1 hydrogen bonds (Table 1 ▸) form helical chains extending along the *c*-axis direction (Fig. 2 ▸). It may be noted that the same bromide ion Br1(*x*, 1 − *y*, *z* − 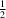) accepts all the identified contacts. These [001] chains pack in the other two dimensions with normal van der Waals contacts (Fig. 3 ▸), in agreement with the results of the Hirshfeld surface analysis (*vide infra*).

## Database survey   

A search of the Cambridge Structural Database (CSD, updated to Dec. 31, 2020; Groom, *et al.*, 2016 ▸) using the fragment **A** yielded 30 hits of which 11 were considered similar to **I**. Among these, (*Z*)-1-[(2*E*)-3,4-diphenyl-2,3-di­hydro-1,3-thia­zol-2-yl­idene]-2-[1-(4-hy­droxy­phen­yl)ethyl­idene]hydra­zinium bromide unknown solvate (CSD refcode BOCROC; Mague, *et al.*, 2014 ▸) and (*E*)-2-[(2-nitro­phen­yl)methyl­idene]-1-[(2*Z*)-4-phenyl-2,3-di­hydro-1,3-thia­zol-2-yl­idene]hydrazinium bromide (NUCLOO; Hassan *et al.*, 2016 ▸) are the closest analogues and another similar compound is 2-{1-[(3,4-diphenyl-1,3-thia­zol-2(3*H*)-yl­idene)hydrazinyl­idene]eth­yl}pyridinium bromide monohydrate (QOCGIA; Akkurt *et al.*, 2014 ▸). Key bond distances and angles for **I** and these three compounds are listed in supplementary Table 1. In the thia­zole ring there is little variation except for the N—C distance *c* in NUCLOO, which is marginally shorter than in the others, possibly due to the nitro­gen atom being unsubstituted. The most noticeable differences occur in the N—C and C=N distances *d* and *e* where the difference between the two is largest in QOCGIA where the absence of the positive charge on the nitro­gen atom bound to the thia­zole ring leads to a greater localization of the π-electron density in the C=N bond.
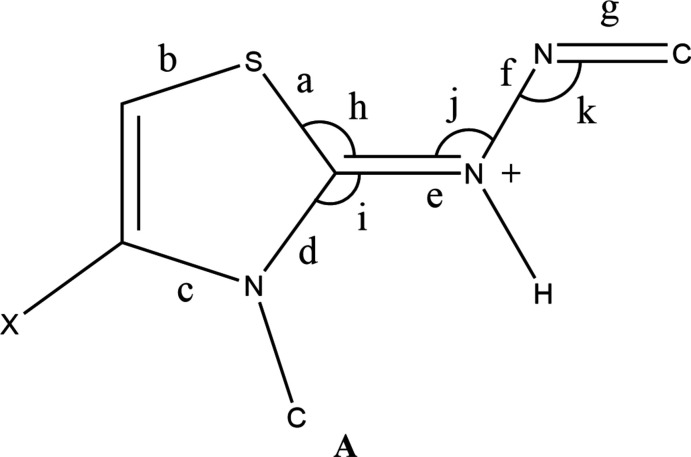


## Hirshfeld surface analysis   

**Figure 4:**
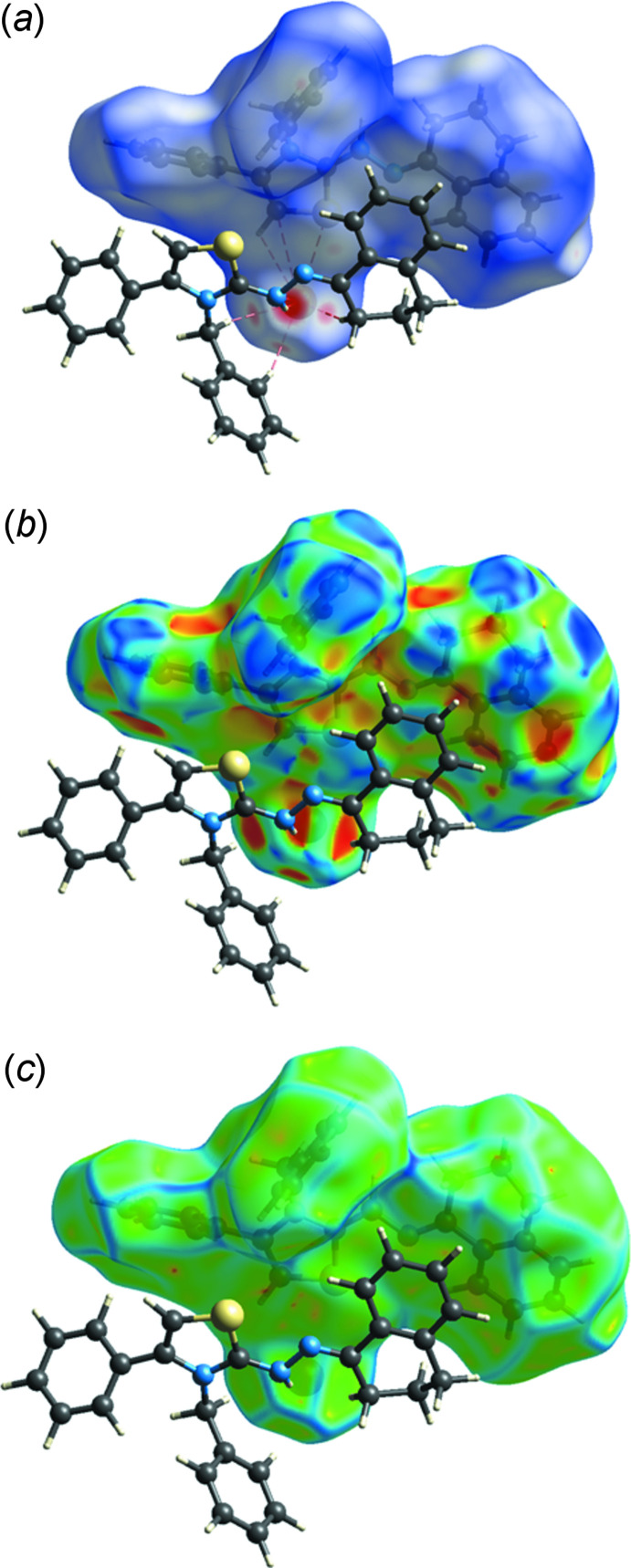
The Hirshfeld surface plotted over (*a*) *d*_norm_ and (*b*) shape and (*c*) curvature indices.

**Figure 5:**
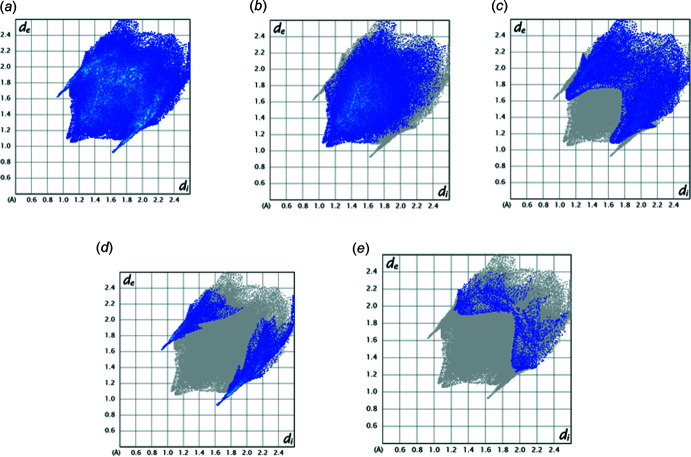
Fingerprint plots showing all (*a*) inter­actions and resolved into (*b*) H⋯H, (*c*) H⋯C/C⋯H, (*d*) Br⋯H/H⋯Br and (*e*) S⋯H/H⋯S contacts.

The Hirshfeld surface for **I** was calculated using *Crystal Explorer17* (Turner *et al.*, 2017 ▸) following the procedures described by Tan *et al.* (2019). Fig. 4 ▸ *a* presents the Hirshfeld surface plotted over *d*_norm_ with a second cation closest to the bromide ion also present, clearly showing the N—H⋯Br and C—H⋯Br inter­actions as well as the S1⋯Br1 short contact (dashed lines). The surface plotted over shape (Fig. 4 ▸ *b*) and curvature indices (Fig. 4 ▸ *c*) do not show much flat surface or evidence for π-stacking inter­actions, in agreement with the results given in Section 3. Fig. 5 ▸ presents fingerprint plots for all inter­molecular inter­actions (*a*) and resolved into all H⋯H contacts (*b*, 51.3%), H⋯C/C⋯H contacts (*c*, 21.9%), Br⋯H/H⋯Br contacts (*d*, 14.1%) and S⋯H/H⋯S contacts (*d*, 3.3%). The N⋯H/H⋯N contacts contribute only 1.3%.

## Synthesis and crystallization   

The title compound was prepared according to our previously reported method (Mohamed *et al.*, 2013 ▸). Mono-crystals of **I** suitable for X-ray diffraction were obtained by recrystallization of the crude product from ethanol solution.

## Refinement   

Crystal data, data collection and structure refinement details are summarized in Table 2 ▸. H atoms attached to carbon were placed in calculated positions (C—H = 0.95–0.99 Å) while that attached to nitro­gen was placed in a location derived from a difference map and its coordinates adjusted to give N—H = 0.91 Å. All were included as riding contributions with isotropic displacement parameters 1.2–1.5 times those of the attached atoms.

## Supplementary Material

Crystal structure: contains datablock(s) global, I. DOI: 10.1107/S2056989021002863/hb7963sup1.cif

Structure factors: contains datablock(s) I. DOI: 10.1107/S2056989021002863/hb7963Isup2.hkl

Geometrical data for title compound and related phases. DOI: 10.1107/S2056989021002863/hb7963sup3.pdf

Supporting information file. DOI: 10.1107/S2056989021002863/hb7963Isup5.cml

CCDC reference: 2071135

Additional supporting information:  crystallographic information; 3D view; checkCIF report

## Figures and Tables

**Table 1:** Hydrogen-bond geometry (Å, °)

*D*—H⋯*A*	*D*—H	H⋯*A*	*D*⋯*A*	*D*—H⋯*A*
N2—H2⋯Br1^i^	0.91	2.63	3.4633 (18)	152
C10—H10*B*⋯Br1^i^	0.99	2.88	3.837 (2)	164
C20—H20*B*⋯Br1^i^	0.99	2.84	3.7560 (19)	155
C26—H26⋯Br1^i^	0.95	2.90	3.730 (2)	146

Symmetry code: (i) x, -y+1, z-{\script{1\over 2}}.

**Table 2:** Experimental details

Crystal data	
Chemical formula	C_26_H_24_N_3_S^+^·Br^−^
*M*_r_	490.45
Crystal system, space group	Monoclinic, *C* *c*
Temperature (K)	150
*a*, *b*, *c* (Å)	14.5474 (7), 17.8777 (8), 9.0803 (4)
β (°)	108.773 (2)
*V* (Å^3^)	2235.92 (18)
*Z*	4
Radiation type	Mo *K*α
μ (mm^−1^)	1.95
Crystal size (mm)	0.22 × 0.12 × 0.06

Data collection	
Diffractometer	Bruker D8 QUEST PHOTON 3 diffractometer
Absorption correction	Numerical (*SADABS*; Krause *et al.*, 2015 ▸)
*T*_min_, *T*_max_	0.69, 0.89
No. of measured, independent and observed [*I* > 2σ(*I*)] reflections	48057, 6797, 6460
*R*_int_	0.026
(sin θ/λ)_max_ (Å^−1^)	0.715

Refinement	
*R*[*F* ^2^ > 2σ(*F* ^2^)], *wR*(*F* ^2^), *S*	0.023, 0.052, 1.03
No. of reflections	6797
No. of parameters	280
No. of restraints	2
H-atom treatment	H-atom parameters constrained
Δρ_max_, Δρ_min_ (e Å^−3^)	0.56, −0.22
Absolute structure	Parsons *et al.* (2013 ▸)
Absolute structure parameter	0.0130 (18)

Computer programs: *APEX3* and *SAINT* (Bruker, 2020 ▸), *SHELXT* (Sheldrick, 2015*a* ▸), *SHELXL2018/1* (Sheldrick, 2015*b* ▸), *DIAMOND* (Brandenburg & Putz, 2012 ▸) and *SHELXTL* (Sheldrick, 2008 ▸).

## supplementary crystallographic information

### Crystal data

**Table:**

C_26_H_24_N_3_S^+^·Br^−^	*F*(000) = 1008
*M**_r_* = 490.45	*D*_x_ = 1.457 Mg m^−^^3^
Monoclinic, *C**c*	Mo *K*α radiation, λ = 0.71073 Å
*a* = 14.5474 (7) Å	Cell parameters from 9847 reflections
*b* = 17.8777 (8) Å	θ = 2.6–30.5°
*c* = 9.0803 (4) Å	µ = 1.95 mm^−^^1^
β = 108.773 (2)°	*T* = 150 K
*V* = 2235.92 (18) Å^3^	Column, colourless
*Z* = 4	0.22 × 0.12 × 0.06 mm

### Data collection

**Table:**

Bruker D8 QUEST PHOTON 3 diffractometer	6797 independent reflections
Radiation source: fine-focus sealed tube	6460 reflections with *I* > 2σ(*I*)
Graphite monochromator	*R*_int_ = 0.026
Detector resolution: 7.3910 pixels mm^-1^	θ_max_ = 30.6°, θ_min_ = 2.6°
φ and ω scans	*h* = −20→20
Absorption correction: numerical (*SADABS*; Krause *et al.*, 2015)	*k* = −25→25
*T*_min_ = 0.69, *T*_max_ = 0.89	*l* = −12→12
48057 measured reflections	

### Refinement

**Table:**

Refinement on *F*^2^	Secondary atom site location: difference Fourier map
Least-squares matrix: full	Hydrogen site location: mixed
*R*[*F*^2^ > 2σ(*F*^2^)] = 0.023	H-atom parameters constrained
*wR*(*F*^2^) = 0.052	*w* = 1/[σ^2^(*F*_o_^2^) + (0.0262*P*)^2^] where *P* = (*F*_o_^2^ + 2*F*_c_^2^)/3
*S* = 1.03	(Δ/σ)_max_ < 0.001
6797 reflections	Δρ_max_ = 0.56 e Å^−^^3^
280 parameters	Δρ_min_ = −0.22 e Å^−^^3^
2 restraints	Absolute structure: Parsons *et al.* (2013)
Primary atom site location: dual	Absolute structure parameter: 0.0130 (18)

### Special details

**Table:**

Geometry. All esds (except the esd in the dihedral angle between two l.s. planes) are estimated using the full covariance matrix. The cell esds are taken into account individually in the estimation of esds in distances, angles and torsion angles; correlations between esds in cell parameters are only used when they are defined by crystal symmetry. An approximate (isotropic) treatment of cell esds is used for estimating esds involving l.s. planes.
Refinement. Refinement of F^2^ against ALL reflections. The weighted R-factor wR and goodness of fit S are based on F^2^, conventional R-factors R are based on F, with F set to zero for negative F^2^. The threshold expression of F^2^ > 2sigma(F^2^) is used only for calculating R-factors(gt) etc. and is not relevant to the choice of reflections for refinement. R-factors based on F^2^ are statistically about twice as large as those based on F, and R- factors based on ALL data will be even larger. H-atoms attached to carbon were placed in calculated positions (C—H = 0.95 - 0.99 Å) while that attached to nitrogen was placed in a location derived from a difference map and its coordinates adjusted to give N—H = 0.91 %A. All were included as riding contributions with isotropic displacement parameters 1.2 - 1.5 times those of the attached atoms.

### Fractional atomic coordinates and isotropic or equivalent isotropic displacement parameters (Å^2^)

**Table:**

	*x*	*y*	*z*	*U*_iso_*/*U*_eq_	
Br1	0.44542 (2)	0.28839 (2)	0.58421 (2)	0.02486 (6)	
S1	0.49752 (4)	0.46885 (3)	0.48367 (6)	0.02090 (10)	
N1	0.62223 (12)	0.54541 (10)	0.3655 (2)	0.0209 (3)	
N2	0.55946 (13)	0.59721 (10)	0.3976 (2)	0.0224 (4)	
H2	0.543925	0.639353	0.338574	0.027*	
N3	0.42532 (11)	0.59882 (9)	0.49354 (19)	0.0175 (3)	
C1	0.68896 (14)	0.57005 (11)	0.3140 (2)	0.0200 (4)	
C2	0.74987 (14)	0.51162 (12)	0.2755 (2)	0.0204 (4)	
C3	0.72405 (16)	0.43611 (12)	0.2725 (3)	0.0253 (4)	
H3	0.668268	0.422211	0.298899	0.030*	
C4	0.77838 (18)	0.38168 (12)	0.2319 (3)	0.0292 (5)	
H4	0.760010	0.330612	0.230188	0.035*	
C5	0.86007 (17)	0.40157 (14)	0.1934 (3)	0.0307 (5)	
H5	0.897430	0.364250	0.164362	0.037*	
C6	0.88672 (17)	0.47585 (13)	0.1977 (3)	0.0307 (5)	
H6	0.943027	0.489020	0.172126	0.037*	
C7	0.83287 (15)	0.53211 (12)	0.2386 (3)	0.0247 (4)	
C8	0.86140 (18)	0.61314 (13)	0.2398 (4)	0.0377 (6)	
H8A	0.900626	0.620413	0.169772	0.045*	
H8B	0.901484	0.627820	0.346198	0.045*	
C9	0.77120 (19)	0.66194 (13)	0.1863 (3)	0.0335 (5)	
H9A	0.732936	0.648836	0.077981	0.040*	
H9B	0.790648	0.715091	0.188104	0.040*	
C10	0.70864 (16)	0.65141 (12)	0.2911 (3)	0.0251 (4)	
H10A	0.741830	0.674405	0.393677	0.030*	
H10B	0.646075	0.677709	0.244715	0.030*	
C11	0.49537 (14)	0.56397 (11)	0.4544 (2)	0.0181 (4)	
C12	0.39810 (15)	0.47730 (11)	0.5475 (2)	0.0212 (4)	
H12	0.368071	0.436044	0.579688	0.025*	
C13	0.36779 (14)	0.54826 (10)	0.5468 (2)	0.0174 (4)	
C14	0.28248 (14)	0.57165 (11)	0.5907 (2)	0.0181 (4)	
C15	0.19406 (15)	0.53523 (12)	0.5184 (2)	0.0248 (4)	
H15	0.189962	0.497890	0.442080	0.030*	
C16	0.11274 (16)	0.55376 (13)	0.5583 (3)	0.0313 (5)	
H16	0.052661	0.529488	0.508398	0.038*	
C17	0.11875 (17)	0.60770 (13)	0.6709 (3)	0.0307 (5)	
H17	0.062685	0.620568	0.697431	0.037*	
C18	0.20603 (17)	0.64264 (12)	0.7444 (3)	0.0273 (5)	
H18	0.210020	0.678992	0.822524	0.033*	
C19	0.28772 (16)	0.62514 (11)	0.7051 (3)	0.0214 (4)	
H19	0.347538	0.649539	0.756080	0.026*	
C20	0.40757 (14)	0.68028 (11)	0.4697 (2)	0.0189 (4)	
H20A	0.348715	0.693577	0.496169	0.023*	
H20B	0.394775	0.692002	0.358296	0.023*	
C21	0.49177 (14)	0.72801 (11)	0.5665 (2)	0.0188 (4)	
C22	0.52950 (17)	0.72086 (12)	0.7273 (3)	0.0261 (4)	
H22	0.505130	0.683350	0.778964	0.031*	
C23	0.60270 (18)	0.76845 (15)	0.8124 (3)	0.0329 (5)	
H23	0.627952	0.763814	0.922321	0.039*	
C24	0.63909 (17)	0.82285 (15)	0.7369 (3)	0.0345 (5)	
H24	0.689705	0.855074	0.795090	0.041*	
C25	0.60187 (18)	0.82998 (14)	0.5785 (3)	0.0371 (6)	
H25	0.626501	0.867479	0.527208	0.044*	
C26	0.52811 (19)	0.78259 (12)	0.4922 (3)	0.0287 (5)	
H26	0.502782	0.787744	0.382335	0.034*	

### Atomic displacement parameters (Å^2^)

**Table:**

	*U*^11^	*U*^22^	*U*^33^	*U*^12^	*U*^13^	*U*^23^
Br1	0.03693 (11)	0.01870 (9)	0.02011 (9)	0.00145 (9)	0.01078 (7)	0.00129 (8)
S1	0.0231 (2)	0.0190 (2)	0.0225 (2)	0.00341 (19)	0.01004 (18)	−0.00098 (19)
N1	0.0207 (8)	0.0209 (8)	0.0239 (8)	0.0026 (7)	0.0109 (7)	−0.0022 (7)
N2	0.0229 (8)	0.0213 (9)	0.0274 (9)	0.0018 (7)	0.0144 (7)	−0.0006 (7)
N3	0.0185 (8)	0.0170 (7)	0.0193 (8)	−0.0005 (6)	0.0091 (6)	−0.0005 (6)
C1	0.0196 (9)	0.0208 (9)	0.0195 (9)	0.0018 (8)	0.0061 (7)	−0.0012 (7)
C2	0.0201 (9)	0.0220 (10)	0.0201 (9)	0.0036 (8)	0.0077 (8)	−0.0008 (8)
C3	0.0248 (10)	0.0241 (10)	0.0297 (11)	0.0029 (8)	0.0123 (9)	0.0023 (8)
C4	0.0337 (12)	0.0202 (10)	0.0356 (12)	0.0037 (9)	0.0139 (10)	−0.0005 (9)
C5	0.0297 (12)	0.0287 (11)	0.0356 (12)	0.0090 (9)	0.0130 (10)	−0.0043 (9)
C6	0.0247 (11)	0.0332 (12)	0.0400 (13)	0.0041 (9)	0.0182 (10)	−0.0003 (10)
C7	0.0222 (10)	0.0250 (10)	0.0297 (11)	0.0013 (8)	0.0122 (9)	−0.0002 (9)
C8	0.0313 (13)	0.0271 (12)	0.0657 (18)	−0.0026 (9)	0.0310 (13)	−0.0010 (12)
C9	0.0422 (13)	0.0214 (10)	0.0471 (15)	−0.0008 (10)	0.0288 (12)	0.0040 (10)
C10	0.0252 (11)	0.0198 (10)	0.0342 (12)	0.0015 (8)	0.0149 (9)	−0.0004 (9)
C11	0.0194 (9)	0.0179 (9)	0.0169 (9)	0.0003 (7)	0.0058 (7)	−0.0019 (7)
C12	0.0217 (9)	0.0209 (9)	0.0234 (10)	−0.0016 (8)	0.0105 (8)	−0.0014 (8)
C13	0.0187 (9)	0.0180 (9)	0.0165 (8)	−0.0007 (7)	0.0069 (7)	0.0012 (7)
C14	0.0187 (9)	0.0174 (9)	0.0199 (9)	0.0005 (7)	0.0086 (7)	0.0039 (7)
C15	0.0245 (10)	0.0258 (11)	0.0250 (10)	−0.0049 (8)	0.0094 (8)	−0.0002 (8)
C16	0.0195 (10)	0.0370 (13)	0.0385 (13)	−0.0060 (9)	0.0109 (9)	0.0022 (10)
C17	0.0252 (11)	0.0296 (12)	0.0441 (13)	0.0042 (9)	0.0205 (10)	0.0047 (10)
C18	0.0336 (12)	0.0206 (10)	0.0350 (12)	0.0037 (9)	0.0213 (10)	0.0013 (9)
C19	0.0229 (10)	0.0180 (9)	0.0255 (10)	−0.0007 (8)	0.0110 (8)	0.0014 (8)
C20	0.0209 (9)	0.0167 (9)	0.0209 (9)	0.0008 (7)	0.0092 (8)	0.0008 (7)
C21	0.0203 (10)	0.0177 (9)	0.0203 (9)	0.0013 (7)	0.0094 (8)	−0.0022 (7)
C22	0.0273 (11)	0.0310 (11)	0.0213 (10)	0.0025 (8)	0.0096 (9)	0.0025 (8)
C23	0.0305 (12)	0.0409 (13)	0.0233 (11)	0.0029 (10)	0.0031 (9)	−0.0048 (10)
C24	0.0307 (12)	0.0338 (13)	0.0373 (13)	−0.0052 (10)	0.0088 (10)	−0.0148 (10)
C25	0.0446 (15)	0.0309 (13)	0.0370 (13)	−0.0187 (11)	0.0151 (11)	−0.0066 (10)
C26	0.0398 (13)	0.0245 (11)	0.0225 (11)	−0.0089 (9)	0.0110 (10)	−0.0032 (8)

### Geometric parameters (Å, º)

**Table:**

S1—C11	1.720 (2)	C10—H10B	0.9900
S1—C12	1.729 (2)	C12—C13	1.342 (3)
N1—C1	1.283 (3)	C12—H12	0.9500
N1—N2	1.396 (2)	C13—C14	1.480 (3)
N2—C11	1.341 (3)	C14—C19	1.396 (3)
N2—H2	0.9100	C14—C15	1.402 (3)
N3—C11	1.337 (2)	C15—C16	1.384 (3)
N3—C13	1.419 (2)	C15—H15	0.9500
N3—C20	1.483 (2)	C16—C17	1.388 (3)
C1—C2	1.483 (3)	C16—H16	0.9500
C1—C10	1.510 (3)	C17—C18	1.379 (3)
C2—C3	1.399 (3)	C17—H17	0.9500
C2—C7	1.402 (3)	C18—C19	1.382 (3)
C3—C4	1.377 (3)	C18—H18	0.9500
C3—H3	0.9500	C19—H19	0.9500
C4—C5	1.388 (3)	C20—C21	1.519 (3)
C4—H4	0.9500	C20—H20A	0.9900
C5—C6	1.381 (3)	C20—H20B	0.9900
C5—H5	0.9500	C21—C26	1.384 (3)
C6—C7	1.397 (3)	C21—C22	1.391 (3)
C6—H6	0.9500	C22—C23	1.387 (3)
C7—C8	1.506 (3)	C22—H22	0.9500
C8—C9	1.519 (4)	C23—C24	1.389 (4)
C8—H8A	0.9900	C23—H23	0.9500
C8—H8B	0.9900	C24—C25	1.370 (4)
C9—C10	1.525 (3)	C24—H24	0.9500
C9—H9A	0.9900	C25—C26	1.394 (3)
C9—H9B	0.9900	C25—H25	0.9500
C10—H10A	0.9900	C26—H26	0.9500
			
C11—S1—C12	89.42 (10)	N2—C11—S1	121.16 (15)
C1—N1—N2	118.09 (17)	C13—C12—S1	112.95 (15)
C11—N2—N1	111.65 (17)	C13—C12—H12	123.5
C11—N2—H2	121.5	S1—C12—H12	123.5
N1—N2—H2	118.8	C12—C13—N3	112.01 (17)
C11—N3—C13	112.19 (16)	C12—C13—C14	124.63 (17)
C11—N3—C20	122.06 (16)	N3—C13—C14	123.33 (16)
C13—N3—C20	125.56 (15)	C19—C14—C15	119.26 (19)
N1—C1—C2	115.06 (19)	C19—C14—C13	122.95 (18)
N1—C1—C10	125.46 (19)	C15—C14—C13	117.71 (18)
C2—C1—C10	119.48 (18)	C16—C15—C14	119.9 (2)
C3—C2—C7	119.61 (19)	C16—C15—H15	120.0
C3—C2—C1	120.48 (18)	C14—C15—H15	120.0
C7—C2—C1	119.89 (19)	C15—C16—C17	120.2 (2)
C4—C3—C2	120.9 (2)	C15—C16—H16	119.9
C4—C3—H3	119.6	C17—C16—H16	119.9
C2—C3—H3	119.6	C18—C17—C16	120.1 (2)
C3—C4—C5	119.9 (2)	C18—C17—H17	120.0
C3—C4—H4	120.1	C16—C17—H17	120.0
C5—C4—H4	120.1	C17—C18—C19	120.4 (2)
C6—C5—C4	119.7 (2)	C17—C18—H18	119.8
C6—C5—H5	120.2	C19—C18—H18	119.8
C4—C5—H5	120.2	C18—C19—C14	120.1 (2)
C5—C6—C7	121.6 (2)	C18—C19—H19	119.9
C5—C6—H6	119.2	C14—C19—H19	119.9
C7—C6—H6	119.2	N3—C20—C21	113.39 (16)
C6—C7—C2	118.4 (2)	N3—C20—H20A	108.9
C6—C7—C8	121.2 (2)	C21—C20—H20A	108.9
C2—C7—C8	120.42 (19)	N3—C20—H20B	108.9
C7—C8—C9	110.03 (19)	C21—C20—H20B	108.9
C7—C8—H8A	109.7	H20A—C20—H20B	107.7
C9—C8—H8A	109.7	C26—C21—C22	119.5 (2)
C7—C8—H8B	109.7	C26—C21—C20	118.49 (19)
C9—C8—H8B	109.7	C22—C21—C20	121.89 (19)
H8A—C8—H8B	108.2	C23—C22—C21	120.1 (2)
C8—C9—C10	110.9 (2)	C23—C22—H22	120.0
C8—C9—H9A	109.5	C21—C22—H22	120.0
C10—C9—H9A	109.5	C22—C23—C24	120.1 (2)
C8—C9—H9B	109.5	C22—C23—H23	120.0
C10—C9—H9B	109.5	C24—C23—H23	120.0
H9A—C9—H9B	108.0	C25—C24—C23	119.9 (2)
C1—C10—C9	112.50 (18)	C25—C24—H24	120.1
C1—C10—H10A	109.1	C23—C24—H24	120.1
C9—C10—H10A	109.1	C24—C25—C26	120.4 (2)
C1—C10—H10B	109.1	C24—C25—H25	119.8
C9—C10—H10B	109.1	C26—C25—H25	119.8
H10A—C10—H10B	107.8	C21—C26—C25	120.0 (2)
N3—C11—N2	125.43 (18)	C21—C26—H26	120.0
N3—C11—S1	113.41 (14)	C25—C26—H26	120.0
			
C1—N1—N2—C11	−178.27 (18)	C11—S1—C12—C13	−0.65 (16)
N2—N1—C1—C2	−177.26 (17)	S1—C12—C13—N3	0.0 (2)
N2—N1—C1—C10	2.7 (3)	S1—C12—C13—C14	177.93 (15)
N1—C1—C2—C3	10.0 (3)	C11—N3—C13—C12	0.9 (2)
C10—C1—C2—C3	−170.0 (2)	C20—N3—C13—C12	176.04 (18)
N1—C1—C2—C7	−171.39 (19)	C11—N3—C13—C14	−177.07 (18)
C10—C1—C2—C7	8.6 (3)	C20—N3—C13—C14	−1.9 (3)
C7—C2—C3—C4	−0.8 (3)	C12—C13—C14—C19	125.0 (2)
C1—C2—C3—C4	177.8 (2)	N3—C13—C14—C19	−57.4 (3)
C2—C3—C4—C5	0.1 (3)	C12—C13—C14—C15	−51.8 (3)
C3—C4—C5—C6	0.6 (4)	N3—C13—C14—C15	125.9 (2)
C4—C5—C6—C7	−0.6 (4)	C19—C14—C15—C16	1.5 (3)
C5—C6—C7—C2	−0.2 (4)	C13—C14—C15—C16	178.37 (19)
C5—C6—C7—C8	−178.8 (2)	C14—C15—C16—C17	−0.7 (3)
C3—C2—C7—C6	0.8 (3)	C15—C16—C17—C18	−0.5 (4)
C1—C2—C7—C6	−177.8 (2)	C16—C17—C18—C19	0.9 (4)
C3—C2—C7—C8	179.5 (2)	C17—C18—C19—C14	−0.2 (3)
C1—C2—C7—C8	0.8 (3)	C15—C14—C19—C18	−1.1 (3)
C6—C7—C8—C9	144.2 (2)	C13—C14—C19—C18	−177.76 (19)
C2—C7—C8—C9	−34.4 (3)	C11—N3—C20—C21	−64.4 (2)
C7—C8—C9—C10	58.8 (3)	C13—N3—C20—C21	120.94 (19)
N1—C1—C10—C9	−163.3 (2)	N3—C20—C21—C26	128.1 (2)
C2—C1—C10—C9	16.7 (3)	N3—C20—C21—C22	−55.2 (2)
C8—C9—C10—C1	−50.3 (3)	C26—C21—C22—C23	0.3 (3)
C13—N3—C11—N2	178.69 (19)	C20—C21—C22—C23	−176.3 (2)
C20—N3—C11—N2	3.3 (3)	C21—C22—C23—C24	−0.6 (4)
C13—N3—C11—S1	−1.4 (2)	C22—C23—C24—C25	0.7 (4)
C20—N3—C11—S1	−176.74 (14)	C23—C24—C25—C26	−0.5 (4)
N1—N2—C11—N3	−178.87 (18)	C22—C21—C26—C25	−0.1 (4)
N1—N2—C11—S1	1.2 (2)	C20—C21—C26—C25	176.6 (2)
C12—S1—C11—N3	1.18 (15)	C24—C25—C26—C21	0.2 (4)
C12—S1—C11—N2	−178.90 (18)		

### Hydrogen-bond geometry (Å, º)

**Table:**

*D*—H···*A*	*D*—H	H···*A*	*D*···*A*	*D*—H···*A*
N2—H2···Br1^i^	0.91	2.63	3.4633 (18)	152
C10—H10*B*···Br1^i^	0.99	2.88	3.837 (2)	164
C20—H20*B*···Br1^i^	0.99	2.84	3.7560 (19)	155
C26—H26···Br1^i^	0.95	2.90	3.730 (2)	146

Symmetry code: (i) *x*, −*y*+1, *z*−1/2.

### Comparison of pertinent bond lengths and angles (Å, °)

**Table:**

Metric*	I	BOCROC	NUCLOO	QOCGIA
a	1.720 (2)	1.711 (4)	1.7182 (15)	1.740 (2)
b	1.729 (2)	1.740 (4)	1.7373 (15)	1.735 (3)
c	1.419 (2)	1.417 (5)	1.3974 (19)	1.414 (4)
d	1.337 (2)	1.341 (4)	1.3314 (19)	1.373 (4)
e	1.341 (3)	1.329 (5)	1.328 (2)	1.309 (3)
f	1.396 (2)	1.395 (4)	1.3806 (17)	1.381 (3)
g	1.283 (3)	1.275 (5)	1.280 (2)	1.293 (4)
h	121.16 (15)	123.0 (3)	124.94 (11)	126.51 (19)
i	125.43 (18)	123.8 (3)	122.85 (13)	122.3 (2)
j	111.65 (17)	114.9 (3)	117.49 (12)	109.4 (2)
k	118.09 (17)	115.5 (3)	114.40 (13)	116.0 (2)

*Key is A in Scheme 2.
