# Supplementary material for: Crystal structure of (E)-1-(3-benzyl-5-phenyl-1,3-thia­zol-2-yl­idene)-2-[(E)-1,2,3,4-tetra­hydro­naphthalen-1-yl­idene]hydrazin-1-ium bromide
Source: Acta Crystallogr E Crystallogr Commun. 2021 Mar 23;77(Pt 4):420–3. doi: 10.1107/S2056989021002863 (PMC8025854; doi:10.1107/S2056989021002863)
Supplement: Supplementary file 3 [file e-77-00420-sup3.pdf]

Supplementary materials:

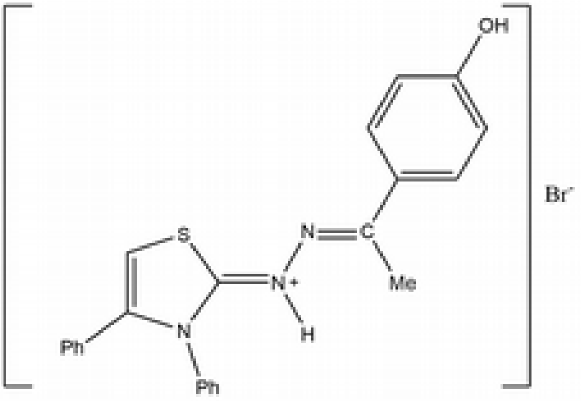

B

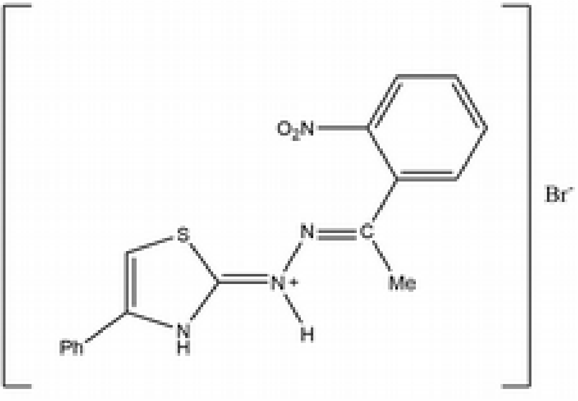

C

B = BOCROC, C = NUCLOO

Comparison of pertinent bond lengths and angles (Å, °).

Metric*	I	BOCROC	NUCLOO	QOCGIA
a	1.720 (2)	1.711 (4)	1.7182 (15)	1.740 (2)
b	1.729 (2)	1.740 (4)	1.7373 (15)	1.735 (3)
c	1.419 (2)	1.417 (5)	1.3974 (19)	1.414 (4)
d	1.337 (2)	1.341 (4)	1.3314 (19)	1.373 (4)
e	1.341 (3)	1.329 (5)	1.328 (2)	1.309 (3)
f	1.396 (2)	1.395 (4)	1.3806 (17)	1.381 (3)
g	1.283 (3)	1.275 (5)	1.280 (2)	1.293 (4)
h	121.16 (15)	123.0 (3)	124.94 (11)	126.51 (19)
i	125.43 (18)	123.8 (3)	122.85 (13)	122.3 (2)
j	111.65 (17)	114.9 (3)	117.49 (12)	109.4 (2)
k	118.09 (17)	115.5 (3)	114.40 (13)	116.0 (2)

*Key is **A** in Scheme 2.
